# QUANTITATIVE AND QUALITATIVE INVESTIGATION OF THREE-DIMENSIONAL GAIT ANALYSIS IN PATIENTS WITH CERVICAL MYELOPATHY

**DOI:** 10.1590/1413-785220243205e278864

**Published:** 2024-10-28

**Authors:** Diara Raiane Santos, Emilly Zatta Pimenta, Thabata Pasquini Soeira, Felipe de Souza Serenza, Mariana Demétrio de Sousa Pontes, Carlos Fernando Pereira da Silva Herrero

**Affiliations:** 1. Universidade de Sao Paulo, Faculdade de Medicina, Hospital das Clínicas de Ribeirao Preto, Departamento de Ortopedia e Anestesiologia, Ribeirao Preto, SP, Brazil.; 2. Universidade de Sao Paulo, Faculdade de Medicina, Hospital das Clínicas de Ribeirao Preto, Departamento de Reabilitação e Desempenho Funcional, Ribeirao Preto, SP, Brazil.

**Keywords:** Gait, Gait Analysis, Myelopathy, Movement, Marcha, Análise da Marcha, Mielopatia, Movimento

## Abstract

Objective: This study aims to describe a kinematic gait assessment protocol and identify its main alterations in individuals with cervical spondylotic myelopathy (CSM) compared to healthy patients. Methods: In total, 14 patients diagnosed with CSM were enrolled and submitted to a three-dimensional gait analysis. The movement of patients was captured with infrared emission cameras that identified tracking markers placed on the lower limbs. Reference positions were used, and the patients walked along a rubberized walkway. The Gait Profile Score (GPS) and Movement Analysis Profile (MAP) were used to analyze variables. Results were subjected to a Student’s t-test at 95% confidence interval. The R Core Team (2016) software was used for statistical analysis, graphically comparing the study results with data from healthy patients. Results: When comparing the kinematic data bilaterally, no statistical differences were found. However, graphical analysis showed changes in the gait of patients with CSM compared to healthy individuals. There were differences in all movements, with a more significant discrepancy in hip and knee flexion and extension, dorsiflexion and plantar flexion, and internal and external hip rotation. Conclusion: We describe a protocol for gait kinematics assessment using GPS and MAP, and we presented the differences in gait kinematics in patients with CSM compared to healthy individuals. ***Level of Evidence II, Prospective study.***

## RESUMO

Objetivo: Descrever um protocolo de avaliação cinemática da marcha e identificar suas principais alterações, em indivíduos com mielopatia espondilótica cervical (MEC), em comparação com pacientes saudáveis. Métodos: 14 pacientes com diagnóstico de MEC foram incluídos e submetidos à análise tridimensional da marcha. Capturamos o movimento dos pacientes com câmeras de emissão infravermelha, que identificaram marcadores de rastreamento posicionados nos membros inferiores. Adquirimos sua posição de referência e os pacientes caminharam por uma passarela emborrachada. As variáveis foram analisadas pelo Gait Profile Score (GPS) e Movement Analysis Profile (MAP), sendo seus resultados analisados pelo teste t de Student com intervalo de confiança de 95%. Utilizamos o software R Core Team (2016) para análise estatística. Comparamos graficamente nossos resultados com dados de pacientes saudáveis. Resultados: Ao comparar os dados cinemáticos bilateralmente, não houve diferenças estatísticas. A análise gráfica evidenciou alterações na marcha do paciente em comparação a indivíduos saudáveis. Houve diferenças em todos os movimentos, com discrepância mais significativa na flexão e extensão do quadril e joelho, dorsiflexão e flexão plantar e rotação interna e externa do quadril. Conclusão: Descrevemos um protocolo de avaliação cinemática da marcha utilizando GPS e MAP e apresentamos as diferenças da marcha de pacientes com MEC, em comparação com indivíduos saudáveis. ***Nível de Evidência II, Estudo prospectivo.***

## INTRODUCTION

 Cervical spondylotic myelopathy (CSM) results from degeneration of the cervical spine. It is characterized by spinal canal narrowing and consequent spinal cord compression, the leading cause of spinal cord dysfunction in adult patients, especially those over 60. ^1^ ^,^ ^2^ Clinical presentation of CSM may include motor dysfunction, gait disturbance, upper limb paresthesia, lower limb weakness or numbness, balance problems, neck pain, and stiffness. ^3^

 Previous studies have shown that most patients diagnosed with CSM have significant dysfunctions at some point in the gait cycle compared to healthy individuals. ^4^ Some of the findings already reported in the literature include slower gait speed, reduced step and gait length, and increased stride width. ^5^ Some researchers have also observed a greater range of motion (ROM) of the ankle and a lower ROM of the knee. ^5^

 Gait analysis in patients with CSM allows a better understanding of gait biomechanics. ^6^ It may provide specific parameters that can be analyzed and compared between the pre and postoperative period, acknowledging a more detailed analysis of the muscle activity and limb movement during all gait phases. ^6^

 The gait parameters of patients with CSM still need to be well established in the literature due to their variability and lack of a defined pattern. ^7^ ^,^ ^8^ ^,^ ^9^ Previous studies have analyzed the gait of patients with CSM. ^8^ ^,^ ^9^ ^,^ ^10^ However, there is a gap in the detailed quantitative and qualitative assessment of the functional performance of these individuals at all phases of the gait cycle. Therefore, this study aimed to identify the main changes in the gait parameters of patients diagnosed with CSM, as well as describe a protocol for kinematic gait assessment. 

## MATERIALS AND METHODS

This prospective study was conducted at the Rehabilitation Center of our institution, received approval by the Institutional Research Ethics Committee (4,026,013) and followed the prerogatives regarding the parameters of research with human beings. The patients who agreed to participate in the study signed an informed consent form.

Patients recruited at the Rehabilitation Center of the Hospital were individuals with CSM diagnoses who were in the evaluation process before surgical treatment. Inclusion criteria were individuals of both sexes, over 18 years of age, who were clinically diagnosed with CSM and confirmed by magnetic resonance imaging, and who agreed to participate by signing an informed consent form. The exclusion criteria included the presence of severe cardiac and respiratory disorders, neurological diseases concomitant with CSM, and/or symptomatic musculoskeletal conditions that could impact gait performance.

Before the surgical procedure, the subjects underwent a three-dimensional gait analysis in a laboratory specialized in gait analysis (LGA). The LGA presents eight infrared emission cameras (Qualisys model Oqus 300) positioned and fixed at approximately 2.6 m from the ground to capture body movement by placing reflective markers on the skin.

**Figure 1.**
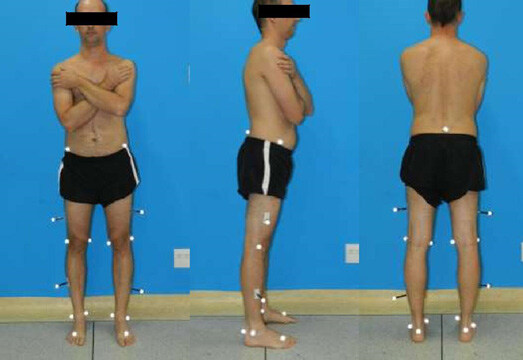
Placement of reflective reference and tracking markers at anatomical points.

 In total, two types of markers were placed tracking markers and reference markers, which allowed building the biomechanical model by the segments’ length and the joint axes’ location. ^11^ ^,^ ^12^ The constructed elements were the pelvis, thigh, leg, and foot. The anatomical points where markers were placed included the anterosuperior iliac spine, the midpoint of the sacrum between the posterosuperior iliac spines, the lateral femoral epicondyles, the lateral malleolus, the calcaneal tuberosity, and the center between the II and III metatarsal bones, previously identified via palpation, as shown in Figure 1 . The purpose of tracking markers is to follow the trajectory of each segment during movement. ^11^ ^,^ ^12^ Tracking markers were used in the thigh, leg, and foot segments, fixed laterally at the midpoint of the thigh and leg ( Figure 1 ). 

To identify the segments, the reference points were obtained by instructing participants to remain in an orthostatic position, with their feet parallel in the center of the walkway for 5 seconds. After this acquisition, the reference markers were removed, continuing with tracking markers. Then, the subjects were instructed to walk barefoot at a comfortable speed along the rubberized walkway. The patients walked at least five times along the entire walkway and performed eight to twelve steps each turn, according to their step size.

 The tracking markers were used to obtain nine kinematic variables and generate the Gait Profile Score (GPS) to facilitate the understanding of the gait analysis. ^13^ From the division of the GPS results, the Gait Variable Score (GVS) was obtained, an index that measures the variable deviation of a normal gait. ^14^ Lastly, the Movement Analysis Profile (MAP) was created from the GVS results. The MAP describes the magnitude of deviation of the nine individual variables estimated over the gait cycle, which shows which variables contribute to altered GPS. ^15^

The statistical data processing was performed using R (R Core Team, 2016), a language and environment for statistical computing, provided by the R Foundation for Statistical Computing, Vienna, Austria. The results were compared using the Student’s t-test for paired samples at a 95% confidence interval for the differences between the sides. The mean values obtained from each evaluation were plotted on a graph to compare with those obtained from healthy individuals.

## RESULTS

Our sample consisted of 14 patients diagnosed with CSM with a mean age of 56 ± 14.85, with 78.57% male (3 women and 11 men). The mean weight was 76.6 Kg ± 14.10 Kg, and the mean height was 1.66 cm ± 0.08 cm. The control group was composed of 19 volunteers with a mean age of 32 years (SD 6.69), mean weight of 61.2 (SD 13.19) and height of 1.70 cm (SD 10.1).

Table 1 shows the results obtained from the kinematic evaluation GPS and MAP. When we compare the means between the sides (right and left), the only joint evaluated that presented a statistically significant difference was the ankle joint (p-value: 0.0204) with a confidence interval from −4.44 to −0.33 ( Table 1 ). 

**Figure 2.**
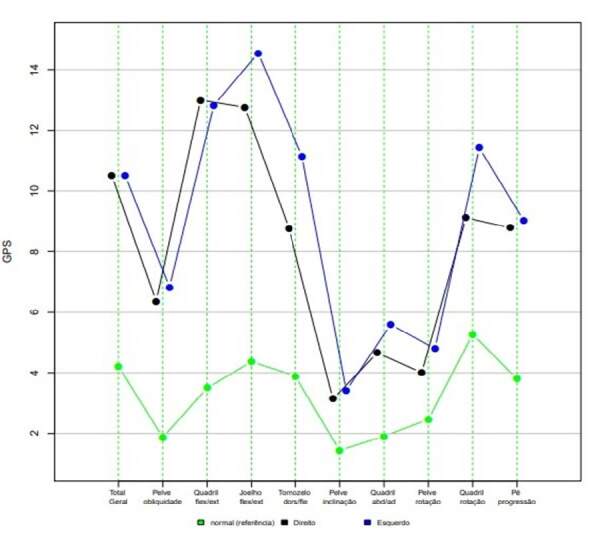
Gait Profile Score and Movement Analysis Profile of individuals with cervical spondylotic myelopathy compared to healthy individuals.

Figure 2 compares the GPS and MAP data obtained in our sample of CSM patients with the GPS and MAP values of a sample of healthy individuals. From the graphic, we identified differences in all the movements studied by kinematic evaluation, with a more significant discrepancy in hip flexion and extension, knee flexion and extension, ankle dorsiflexion and plantar flexion, and internal and external hip rotation ( Figure 2 ). 

**Table 1.** Gait Profile Score and Movement Analysis Profile values of individuals with cervical spondylotic myelopathy and healthy individuals.

Variable*	Right		Left		P value	Confidence Interval 95%		Healthy individuals
			
	Mean	Standard Deviation	Mean	Standard Deviation		Inferior Limit	Upper Limit	
				
G	10.51	2.7872	10.51	2.7872	NaN	NaN	NaN	4.21
PO	6.36	3.891	6.83	4.7155	0.4337	−1.72	0.78	1.87
HFE	13	4.9047	12.83	5.3324	0.8138	−1.33	1.66	3.5
KFE	12.76	4.4356	14.55	5.355	0.1628	−4.4	0.82	4.37
ADPF	8.76	2.9999	11.14	2.589	0.0264	−4.44	−0.33	3.89
PT	3.14	1.3039	3.41	1.4509	0.116	−0.63	0.08	1.44
HAA	4.66	1.6082	5.59	2.9295	0.2226	−2.51	0.64	1.91
PR	4.01	2.7065	4.8	3.0339	0.2102	−2.08	0.5	2.46
HIER	9.11	4.621	11.42	6.4778	0.3173	−7.09	2.48	5.27
F	8.79	4.8826	9.03	8.0509	0.934	−6.41	5.93	3.8

*G = general; PO = pelvic obliquity; HFE = hip flexion and extension; KFE = knee flexion and extension; ADPF = ankle dorsiflexion and plantar flexion; PT = pelvic tilt; HAA = hip adduction and abduction; PR = pelvic rotation; HIER = hip internal and external rotation; F = foot progression.

## DISCUSSION

 Several studies have shown that individuals diagnosed with CSM present alterations during the gait cycle compared to healthy individuals due to spinal cord compression. ^4^ ^,^ ^10^ Nevertheless, typical signs and symptoms of CSM are pain in the neck, shoulder, and subscapular areas; numbness or tingling in the upper extremities; motor weakness in the upper or lower extremities; sensory changes in the lower extremities; and gait disturbances represented most commonly by a spastic gait. ^16^ On the other hand, subtle or unusual gait presentations can make the diagnosis of CSM challenging. Thus, three-dimensional gait analysis can be a helpful tool by providing detailed data on the biomechanics and gait impairment in patients with CSM. ^17^ Therefore, our study aimed to analyze the kinematic changes during gait in individuals diagnosed with CSM before the surgical treatment and to describe a gait assessment protocol. 

 This study, by assessing gait of 14 patients with CSM, identified the main kinematic gait alterations and presented an assessment protocol based on three-dimensional gait analysis using specific tools (GPS and MAP). It has been reported that this analysis is important for a better prognosis of CSM after surgery and the prevention of possible falls related to gait impairment, consequently maintaining a good quality of life for patients. ^18^

 GPS consists of nine main kinematic variables that a single number can represent. Its measurement is presented in degrees, and higher values point to greater deviations from a gait considered normal. ^13^ We chose this tool because its results are easy to interpret, and the minimal clinically detectable difference is 1.6°. Thus, angular changes considered clinically undetectable could show statistically significant differences. 

 Additionally, the GPS can be decomposed to provide the Gait Variable Score (GVS). The GVS represents parameters that estimate the gait deviation variation. From the GVS, the Movement Analysis Profile (MAP) is created to describe the magnitude of the deviation of the nine individual variables estimated via the gait cycle, thus providing a view of which variables contribute to a high GPS. ^14^ Thus, we chose MAP due to its capacity of providing additional helpful information to the GPS. To support the value of these tools in analyzing gait parameters, we compared the results found on both sides. Then, except for the ankle joint (p = 0.0204), our results regarding the GPS and the MAP did not show statistically significant differences when comparing both sides. The interpretation of our results suggests a symmetry in the findings on both sides, both statistical and clinical since the minimum difference clinically detectable by GPS is only 1.6°, as mentioned above. 

The graphic comparison of GPS and MAP values obtained from our sample of patients with CSM with the values of individuals considered healthy showed a difference in all parameters studied, with a more significant discrepancy in hip flexion and extension, flexion and extension knee, dorsiflexion and plantar flexion of the ankle, and internal and external rotation of the hip. The interpretation of our results suggests that, although we did not find a statistically significant difference between the sides, the GPS and the MAP allowed us to identify changes in the gait kinematics of patients with CSM.

Our study shows some limitations. Firstly, we highlight the small sample size. As this assessment is not part of the patient’s preoperative protocol routine, we obtained authorization from a small portion of individuals operated in our services during the study period. Secondly, this study lacks a statistical comparison of our results with the findings of individuals considered healthy since we did not have access to the detailed data of the patients involved in the study from which we acquired the values. However, we believe the graphical difference was substantial in all segments studied, reinforcing the importance of evaluating gait kinematics in patients with CSM. Finally, we believe that the increased age of patients can contribute to changes found in the assessment of individuals’ gait, which makes room for a new study proposal. Since subtle changes may occur between the gait of patients with CSM and patients with advanced age, detailed assessment techniques may be extremely relevant.

Thus, we were able to design a protocol for kinematic gait assessment of patients with CSM, identifying possible changes in the gait pattern of these patients. Based on these findings, studies can be developed, including a kinematic gait assessment in the postoperative period, which can serve as an additional tool in evaluating the effectiveness of surgical treatment.

## CONCLUSION

From the study, it was possible to describe a protocol for gait kinematics assessment using GPS and MAP to identify the main alterations, in addition to presenting the differences in gait kinematics of patients with CSM compared to healthy individuals.
